# Factors Associated With Restarting Androgenic Anabolic Steroids After Cessation in Men With Infertility: A Retrospective Analysis

**DOI:** 10.7759/cureus.41134

**Published:** 2023-06-29

**Authors:** Josh White, Armin Ghomeshi, Nicholas A Deebel, David T Miller, Farah Rahman, Greeshma Venigalla, Max Sandler, Ana Tomlinson, Ranjith Ramasamy

**Affiliations:** 1 Urology, University of Miami, Miami, USA; 2 Urology, Florida International University, Herbert Wertheim College of Medicine, Miami, USA; 3 Urology, Atrium Health Wake Forest Baptist, Winston-Salem, USA; 4 Urology, Univeristy of Pittsburgh, Pittsburgh, USA

**Keywords:** men’s health, andrology, ivf, fertility, anabolic steroid abuse

## Abstract

Introduction

The use of androgenic anabolic steroids (AAS) negatively affects male fertility by disrupting hormone release and reducing testosterone levels. Despite this, many men using steroids are unaware of fertility-related consequences. We aimed to determine the factors associated with AAS resumption during fertility treatment, specifically focusing on the duration, age, and dosage of AAS use prior to treatment. Our study, the first of its kind, investigated risk factors for resuming AAS following fertility assessment.

Methods

We conducted a retrospective review of adult men diagnosed with infertility due to chronic AAS use between 2012 and 2022 at the University of Miami. The study included men with azoospermia or severe oligospermia who were instructed to stop using AAS. Excluded were those who underwent orchiectomy for benign or malignant conditions. We collected data on demographic characteristics, AAS route details, fertility treatments, and AAS resumption. We hypothesized that risk factors for restarting AAS would include duration of AAS use, type of AAS, pre-treatment testosterone levels, and increased age.

Results

We identified 94 men with infertility caused by AAS use. Among them, 31 (33.0%) resumed AAS therapy within eight months after cessation. The median age of men who restarted AAS was 40 years. Those who resumed AAS had used it for a longer duration prior to fertility assessment compared to those who did not (60 months vs. 17 months, respectively). However, we found no statistically significant differences in age, duration of AAS use, AAS administration details, or serum testosterone levels at the time of initial assessment.

Conclusion

In conclusion, most men seeking fertility assessment due to AAS abuse did not resume testosterone therapy. However, those who did restart AAS had a longer history of AAS use. Future high-quality prospective studies are needed to better understand the risk factors associated with resuming AAS in male infertility caused by anabolic steroids.

## Introduction

An estimated 2.2 million patients received treatment with androgenic anabolic steroids (AAS) in the year 2013 [1]. AAS use has significant and potentially long-lasting impacts on male fertility due to the disruption of the hypothalamus-pituitary-adrenal axis. AAS exhibits negative feedback on the pituitary, leading to suppressed follicle-stimulating hormone (FSH) and luteinizing hormone (LH) release, thereby resulting in decreased levels of intratesticular testosterone (T) and suppression of spermatogenesis [2]. In normozoospermic men who receive AAS, it has been shown that around 98% will develop oligospermia or azoospermia [3-5]. Despite these deleterious effects, patients are often not properly counseled before the initiation of AAS. Studies report that fewer than half of patients were aware of the effects of AAS on fertility [6]. Alarmingly, physicians prescribing the medication are also not fully aware of the potential impact on fertility. In a study by Ko et. al. 25% of urologists believe that AAS use improves male fertility [7]. Studies report that around 7% of patients seeking fertility assessment will be on AAS [8]. 

Discontinuing AAS use is the first step in the management of patients with male factor infertility secondary to anabolic steroid abuse. Recovery of spermatogenesis after AAS cessation occurs is reported to occur in 64-100% of men within 12 months [4,9,10]. Thus, there are a significant number of patients with persistent abnormal sperm production. Treatments have been used to expedite spermatogenesis, which includes the use of human chorionic gonadotropin (hCG) in combination with FSH and/or selective estrogen receptor modulators [9,11]. However, some men will never regain normal spermatogenesis [9,11]. Previous studies have demonstrated that, after discontinuing AAS, patients have withdrawal symptoms, including transient flu-like symptoms, depression, decreased sex drive, fatigue, muscle pain, joint pain, headaches, and cravings to resume steroids [12-14]. To alleviate these symptoms, medical treatment with fluoxetine, hCG, and clomiphene has been utilized with some success [13,15]. However, for AAS users, it is estimated that 30% will develop dependence [16]. These symptoms likely contribute to a return to AAS use even in those patients seeking fertility treatment.

Shared decision-making with men during their fertility care can have important implications for their decision to restart AAS. Therefore, our objective was to determine if there are identifiable risk factors that lead to an increased likelihood of AAS resumption while undergoing fertility treatment. We hypothesized that time on AAS, age, and dosing of AAS before treatment would be correlated with resuming AAS. To our knowledge, this is the first study to evaluate potential risk factors for resuming AAS following treatment for AAS-associated infertility.

## Materials and methods

A retrospective chart review was performed to identify patients who presented to the University of Miami Men’s Health Department with possible male factor infertility secondary to AAS abuse between 2012 and 2022. Utilizing the University of Miami Health electronic medical record database, we identified 189 patients. We included men aged 18-75 years old, who received treatment in the men’s health clinic at the University of Miami and patients placed on clomiphene citrate and/or hCG following the initial evaluation. Both patients taking prescribed steroids through a provider and those using steroids recreationally were included. impact Those with a history of pelvic radiation, chemotherapy, or radical orchiectomy were excluded. After selection, a total of 94 participants were included in this study.

Data extracted from the electronic medical record included demographics, the dosage of AAS, the route of administration, and the duration of time on AAS. Additionally, we examined serial labs to monitor changes in sperm concentration, FSH, LH, and serum testosterone level (T) as a result of clomiphene and/or hCG therapy. We determined whether the patient had restarted AAS-based patient self-reporting and prescription records. A Mann-Whitney U test was performed on continuous variables, with a p <0.05 defined as statistically significant. All statistical analyses and visualizations were conducted in SAS Studio (Version 5.2).

## Results

A total of 94 men with azoospermia or severe oligospermia secondary to anabolic steroid use were identified, with a median age of 38.5 years (interquartile range (IQR): 33.2-45). A summary of demographic and laboratory values at the time of initial assessment can be viewed in Table 1. Of these 94 men, 31 (33.0%) restarted AAS therapy. These men restarted AAS therapy within eight months (IQR: 6.5-18.0) of cessation. Men who restarted AAS had a median age of 40 years (IQR: 34.5-44.5). Patients that restarted AAS therapy were on AAS for a longer duration prior to fertility assessment compared with those who did not resume (60 months IQR: 27-126 vs. 17 months IQR: 4-60, respectively). Patients who resumed AAS therapy had a median decrease in serum testosterone compared to baseline after starting hCG and/or clomiphene of 234 ng/dL (IQR: -531-6.5).

**Table 1:** Study demographics at the time of initial assessment IQR: Interquartile range, AAS: androgenic anabolic steroids, Conc.: concentration, n: # of participants

Study Demographics		Total (n=94)
Median Age (years) (IQR)		38.5 (33.2-45)
Median Serum Total Testosterone (ng/dL)		414 (239-742)
Median Time on AAS (months) (IQR)		48 (12-20)
Route of AAS	Injection	32 (34%)
	Oral	15 (16%)
	Gel	3 (3%)
	Testopel	2 (2%)
	Unknown	42 (45%)
Median Sperm Conc. (million/cc) (IQR)		0.5 (0-3.5)

We did not identify any statistically significant differences between age, duration of time on AAS therapy, pre-assessment AAS route, and dosing, as well as the serum testosterone at the time of initial assessment (Table 2).

**Table 2:** Differences in baseline characteristics, serum testosterone, and sperm concentration between men who restarted AAS and those that did not restart AAS following fertility assessment IQR: Interquartile range, AAS: androgenic anabolic steroids, Conc.: concentration, cc: cubic centimeter, n: # of participants

Study Demographics		Restarted AAS (n=31)	Not Restarted AAS (n=63)	P value
Median Age (years) (IQR)		40 (33-45)	38 (33-45)	0.9999
Median Serum Total Testosterone (ng/dl) (IQR)		417 (226-664)	450 (316-747)	0.7481
Median Time on AAS (months) (IQR)		61 (24-24-135)	24 (12-72)	0.0548
Route of AAS, n(%)	Injection	10 (32.3)	22 (34.9)	0.4167
	Oral	7 (22.5)	8 (12.7)	
	Gel	2 (6.5)	1 (1.6)	
	Testopel	1 (3.2)	1 (1.6)	
	Unknown	11 (35.5)	31 (49.2)	
Median Sperm Conc. (million/cc) (IQR)		0.68 (0-3.6)	0.30 (0-3.4)	0.6193

## Discussion

**Figure 1:**
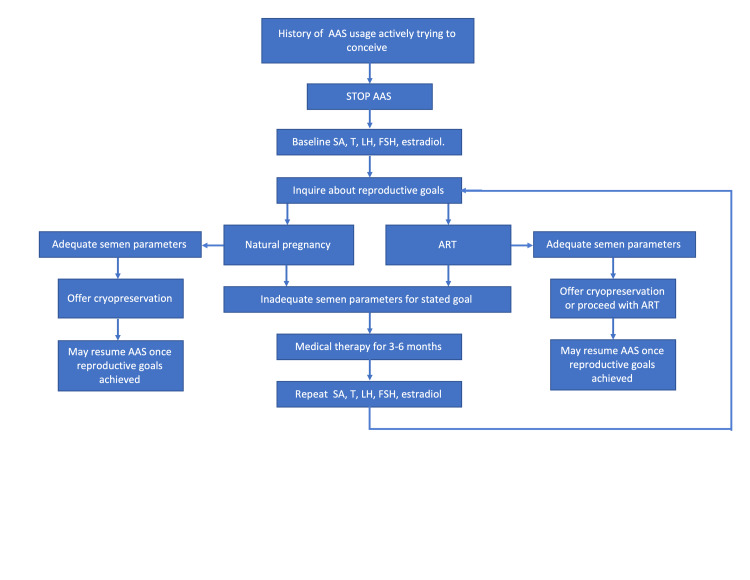
Algorithm for the treatment of androgenic anabolic steroids (AAS)-induced infertility SA, semen analysis; T, testosterone; LH, luteinizing hormone; FSH, follicle stimulating hormone; hCG, human chorionic gonadotropin; ART, assisted reproductive techniques

The use of AAS is a common etiology for secondary male factor infertility. While the treatment of this, consisting of cessation of anabolic steroids and utilization of alternative agents, is well established, data regarding the likelihood of resumption after fertility evaluation are sparse. Identification of predictive factors for resuming AAS use will help clinicians appropriately engage their patients in the shared decision-making process and provide more effective longitudinal care. A proposed algorithm in men with infertility secondary to AAS is shown in Figure 1.

While our study failed to identify statistically significant predictors for relapse in AAS use, there are a myriad of reasons a patient may decide to prematurely resume the use of their AAS therapy. Although alternative therapies, such as clomiphene and hCG, are effective in restoring T to goal ranges, mixed reports are debating the improvement in testosterone deficiency symptoms between these alternative therapies compared to exogenous AAS [17,18]. Patients accustomed to symptomatic improvement from AAS may therefore lack satisfaction with alternative therapies and be inclined to prioritize symptomatic improvement over fertility risks from exogenous therapy. Additionally, partner conditions necessitating the use of in vitro fertilization (IVF) with intracytoplasmic sperm injection (ICSI) may be present. While patients in our series were extensively counseled that AAS use can lead to azoospermia, it is possible oligozoospermic patients were able to provide a semen analysis suitable for IVF/ICSI and elected to resume AAS therapy.

A possible predictor may be the concomitant presence of mental health illnesses, such as anxiety, depression, and variations of body dysmorphic or personality disorders. For instance, the work by Piacentino et al. examined various psychopathologic metrics between nonanabolic steroid users and those testing positive for anabolic steroid use. Patients testing positive for steroids showed higher Hypomania Checklist-32 scores, which in turn were predictive for anabolic androgenic steroid use [19]. Similarly, other works have shown that the presence of personality disorders (narcissistic, antisocial, and borderline) may also be predictive of exogenous steroid use [19-21]. Finally, connections have been made between major depressive disorder, generalized anxiety disorder, eating disorders, and anabolic androgenic steroid use [19,22-24]. While more data are needed to clarify the cause-and-effect relationship between psychopathology and exogenous steroid use, the implication of these relationships warrants further study in prospective models. This will allow for an improved understanding of the male infertility patient experience as clinicians and patients navigate their transition from AAS therapy to alternative therapies.

Patient goals and expectations are important when discussing discontinuing AAS for fertility. While medical therapy with selective estrogen receptor modulators (SERMs) and/or HCG may be effective, certain patients may be committed to IVF/ICSI at the onset for a variety of reasons [3-5]. Furthermore, for patients declining alternative therapy or couples with advanced maternal age, expedited collaboration with female reproductive endocrinologists for IVF/ICSI consideration is prudent.

To the best of our knowledge, this is the first study examining risk factors for relapse of AAS use amongst men with infertility. While this serves as a benchmark for future, prospective work, our study is not without limitations. This was a retrospective analysis, therefore limiting study design and variable inclusion. Most notably this precluded the inclusion of thorough psychiatric history, pharmacotherapy, or concomitant substance abuse. Given the aforementioned relationship between mental health and anabolic steroid use, it would be logical to assume that differences in the presence of these conditions may impact the likelihood of relapse in the use of exogenous testosterone. Future areas of consideration include prospective assessment of partner outcomes to determine the rates of IVF and natural conception in this patient population to more effectively counsel patients.

## Conclusions

AAS use has significant negative effects on male fertility, of which many patients and even providers are unaware. In the present study, potential risk factors that were hypothesized to lead to an increased likelihood of AAS resumption were examined in a cohort of 94 male patients diagnosed with infertility secondary to chronic anabolic-androgenic use. There was a trend that men who resumed AAS therapy had been receiving treatment for longer than patients who remained off AAS therapy. Overall, our study outlines that the likelihood of AAS resumption in patients who chronically use anabolic steroids deserves more clinical attention for the shared patient-provider decision-making about male infertility options. Although it remains to be established whether there are identifiable risk factors for AAS resumption, this is the first study, to our knowledge, that poses this question.

## References

[1] Nguyen CP, Hirsch MS, Moeny D, Kaul S, Mohamoud M, Joffe HV (2015). Testosterone and “age-related hypogonadism”—FDA concerns. N Engl J Med.

[2] MacIndoe JH, Perry PJ, Yates WR, Holman TL, Ellingrod VL, Scott SD (1997). Testosterone suppression of the HPT axis. J Investig Med.

[3] World Health Organization Task Force on Methods for the Regulation of Male Fertility (1996). Contraceptive efficacy of testosterone-induced azoospermia and oligozoospermia in normal men. Fertil Steril.

[4] Gu YQ, Wang XH, Xu D (2003). A multicenter contraceptive efficacy study of injectable testosterone undecanoate in healthy Chinese men. J Clin Endocrinol Metab.

[5] Handelsman DJ, Conway AJ, Howe CJ, Turner L, Mackey MA (1996). Establishing the minimum effective dose and additive effects of depot progestin in suppression of human spermatogenesis by a testosterone depot. J Clin Endocr Metab.

[6] Reshef E, Craig L, Burks HR, Hansen KR, Sindhwani P, Quaas AM (2016). Testosterone (T) use in male partners of couples presenting for the initial infertility evaluation: Prevalence, usage patterns and fertility awareness. Fertil Steril.

[7] Ko EY, Siddiqi K, Brannigan RE, Sabanegh ES Jr (2012). Empirical medical therapy for idiopathic male infertility: A survey of the American Urological Association. J Urol.

[8] Kolettis PN, Purcell ML, Parker W, Poston T, Nangia AK (2015). Medical testosterone: An iatrogenic cause of male infertility and a growing problem. Urology.

[9] Kohn TP, Louis MR, Pickett SM, Lindgren MC, Kohn JR, Pastuszak AW, Lipshultz LI (2017). Age and duration of testosterone therapy predict time to return of sperm count after human chorionic gonadotropin therapy. Fertil Steril.

[10] Liu PY, Swerdloff RS, Anawalt BD (2008). Determinants of the rate and extent of spermatogenic suppression during hormonal male contraception: An integrated analysis. J Clin Endocrinol Metab.

[11] Wenker EP, Dupree JM, Langille GM (2015). The use of hCG-based combination therapy for recovery of spermatogenesis after testosterone use. J Sex Med.

[12] Hochberg Z, Pacak K, Chrousos GP (2003). Endocrine withdrawal syndromes. Endocr Rev.

[13] Malone DA Jr, Dimeff RJ (1992). The use of fluoxetine in depression associated with anabolic steroid withdrawal: A case series. J Clin Psychiatry.

[14] Brower KJ, Eliopulos GA, Blow FC, Catlin DH, Beresford TP (1990). Evidence for physical and psychological dependence on anabolic androgenic steroids in eight weight lifters. Am J Psychiatry.

[15] Kanayama G, Brower KJ, Wood RI, Hudson JI, Pope HG Jr (2010). Treatment of anabolic-androgenic steroid dependence: Emerging evidence and its implications. Drug Alcohol Depend.

[16] Kanayama G, Brower KJ, Wood RI, Hudson JI, Pope HG Jr (2009). Anabolic-androgenic steroid dependence: An emerging disorder. Addiction.

[17] Dadhich P, Ramasamy R, Scovell J, Wilken N, Lipshultz L (2017). Testosterone versus clomiphene citrate in managing symptoms of hypogonadism in men. Indian J Urol.

[18] Ramasamy R, Scovell JM, Kovac JR, Lipshultz LI (2014). Testosterone supplementation versus clomiphene citrate for hypogonadism: An age matched comparison of satisfaction and efficacy. J Urol.

[19] Piacentino D, Sani G, Kotzalidis GD (2022). Anabolic androgenic steroids used as performance and image enhancing drugs in professional and amateur athletes: Toxicological and psychopathological findings. Hum Psychopharmacol.

[20] Porcerelli JH, Sandler BA (1995). Narcissism and empathy in steroid users. Am J Psychiatry.

[21] Yates WR, Perry PJ, Andersen KH (1990). Illicit anabolic steroid use: A controlled personality study. Acta Psychiatr Scand.

[22] Griffiths S, Jacka B, Degenhardt L, Murray SB, Larance B (2018). Physical appearance concerns are uniquely associated with the severity of steroid dependence and depression in anabolic-androgenic steroid users. Drug Alcohol Rev.

[23] Lindqvist AS, Moberg T, Eriksson BO, Ehrnborg C, Rosén T, Fahlke C (2013). A retrospective 30-year follow-up study of former Swedish-elite male athletes in power sports with a past anabolic androgenic steroids use: A focus on mental health. Br J Sports Med.

[24] Ip EJ, Trinh K, Tenerowicz MJ, Pal J, Lindfelt TA, Perry PJ (2015). Characteristics and behaviors of older male anabolic steroid users. J Pharm Pract.

